# CSF inflammatory markers differ in gram-positive versus gram-negative shunt infections

**DOI:** 10.1186/s12974-019-1395-6

**Published:** 2019-01-09

**Authors:** Gwenn L. Skar, David Synhorst, Matthew Beaver, Jessica N. Snowden

**Affiliations:** 1 Department of Pediatrics, University of Nebraska Medical Center, 982162 Nebraska Medical Center, Omaha, NE 68198-2162 USA; 2 Department of Pediatrics, Children’s Mercy Hospital, Kansas City, MO USA; 3 Department of Pediatrics, University of Arkansas for Medical Sciences, Little Rock, AR USA

**Keywords:** Shunt infection, Biomarker, Inflammation, Ventriculoperitoneal catheter, CSF cytokines

## Abstract

**Background:**

Cerebrospinal fluid (CSF) shunt placement is frequently complicated by bacterial infection. Shunt infection diagnosis relies on bacterial culture of CSF which can often produce false-negative results. Negative cultures present a conundrum for physicians as they are left to rely on other CSF indices, which can be unremarkable. New methods are needed to swiftly and accurately diagnose shunt infections. CSF chemokines and cytokines may prove useful as diagnostic biomarkers. The objective of this study was to evaluate the potential of systemic and CSF biomarkers for identification of CSF shunt infection.

**Methods:**

We conducted a retrospective chart review of children with culture-confirmed CSF shunt infection at Children’s Hospital and Medical Center from July 2013 to December 2015. CSF cytokine analysis was performed for those patients with CSF in frozen storage from the same sample that was used for diagnostic culture.

**Results:**

A total of 12 infections were included in this study. Patients with shunt infection had a median C-reactive protein (CRP) of 18.25 mg/dL. Median peripheral white blood cell count was 15.53 × 10^3^ cells/mL. Those with shunt infection had a median CSF WBC of 332 cells/mL, median CSF protein level of 406 mg/dL, and median CSF glucose of 35.5 mg/dL. An interesting trend was observed with gram-positive infections having higher levels of the anti-inflammatory cytokine interleukin (IL)-10 as well as IL-17A and vascular endothelial growth factor (VEGF) compared to gram-negative infections, although these differences did not reach statistical significance. Conversely, gram-negative infections displayed higher levels of the pro-inflammatory cytokines IL-1β, fractalkine (CX_3_CL_1_), chemokine ligand 2 (CCL2), and chemokine ligand 3 (CCL3), although again these were not significantly different. CSF from gram-positive and gram-negative shunt infections had similar levels of interferon gamma (INF-γ), tumor necrosis factor alpha (TNF-α), IL-6, and IL-8.

**Conclusions:**

This pilot study is the first to characterize the CSF cytokine profile in patients with CSF shunt infection and supports the distinction of chemokine and cytokine profiles between gram-negative and gram-positive infections. Additionally, it demonstrates the potential of CSF chemokines and cytokines as biomarkers for the diagnosis of shunt infection.

## Background

Cerebrospinal fluid (CSF) shunts are the most common treatment of hydrocephalus in the USA; however, they are frequently complicated by bacterial infection [1]. Shunt infections are responsible for approximately 2400 admissions and 59,000 hospital days annually in the USA [2]. Bacteria cause the majority of shunt infections, with *Staphylococcus epidermidis* and *Staphylococcus aureus* among the leading causes [3–6]. Apart from staphylococci, *Propionibacterium acnes*, enterococci, and a variety of gram-negative bacteria including *Pseudomonas aeruginosa* and *Escherichia coli* commonly cause shunt infections [3–7].

Shunt infections present unique diagnostic and treatment challenges, since they are biofilm rather than planktonic infections [8]. Bacteria adhere to the catheter and form a biofilm, communities of bacteria which are tolerant to antibiotics and actively avoid immune clearance [9]. Thus, the inflammatory response to biofilm infection is distinct from planktonic infection, such as bacterial meningitis or intracranial abscess [9–12]. Due to their biofilm nature, shunt infection treatment requires both removal of the infected shunt and days or weeks of intravenous antibiotics [13]. With the extensive nature of treatment, it is essential that shunt infections are diagnosed accurately and rapidly.

While newer DNA-based detection methods are being instituted in the clinical microbiology laboratory, the gold standard method to diagnose shunt infection currently relies on isolating a pathogen from CSF culture [13–19]. While DNA sequencing methods for microbiological diagnosis are promising, they also have pitfalls, particularly in the case of shunt infection where many sequenced pathogens are common skin flora. The utility of broad-range polymerase chain reaction (PCR) may be limited by contamination with skin flora during collection and handling as well as antibiotic administration before a diagnosis is made [16]. The only multiplex PCR panel that is currently commercially available for meningitis/encephalitis has a limited spectrum of bacterial organisms, which is too narrow to be diagnostically useful for cerebrospinal fluid shunt infections, where *Staphylococcus epidermidis* and *Staphylococcus aureus* are the predominant organisms that are not included in this panel [3–6].

Traditional CSF culture normally requires 24–48 h to isolate organisms, and some slower growing organisms may require several days of incubation, which can delay appropriate treatment [20]. Additionally, if the patient has received antibiotics prior to the CSF culture, the culture may be falsely negative. In these cases, a clinician must rely on CSF indices such as cell count and differential, glucose, protein levels, and Gram stain, as well as clinical presentation, to determine if an infection is present [19]. A better modality to accelerate and accurately diagnose CSF shunt infections would improve our ability to treat these infections.

Identifying biomarkers in the CSF or serum of patients with shunt infection may improve our ability to diagnose CSF shunt infections. Biomarkers are distinct biochemical, genetic, or molecular substances that characterize infection [20, 21]. There are a few small studies that have evaluated the ability of CSF chemokines and cytokines to characterize different bacterial and viral meningitis. Many studies have examined the utility of using chemokines and cytokines for the diagnosis of meningitis. In early meningeal infection, the pro-inflammatory cytokines interleukin (IL)-8, IL-1, tumor necrosis factor alpha (TNF-α), and IL-6 have been shown to be elevated [22–25]. These mediators are released from CNS resident cells and function to recruit neutrophils and perpetuate the inflammatory response [26, 27]. Once monocytes are recruited, the anti-inflammatory cytokine IL-10 and IL-1β dampen inflammation by inhibiting production of pro-inflammatory cytokines [28].

Several studies have examined the possibility of using chemokines and cytokines as biomarkers for meningitis in the pediatric population. Elevated IL-8, IL-1β, TNF-α, and IL-6 have routinely been shown to be elevated in bacterial meningitis [23–25, 29, 30]. Another study revealed that cytokines could be used to discriminate between meningitis caused by *Streptococcus pneumoniae*, *Neiserria meningitidis*, and *Haemophilus influenzae* based on levels of interferon gamma (IFN-γ), monocyte chemoattractant protein-1 (MCP-1), and the enzyme matrix metallopeptidase-9 (MMP-9) [31].

Studies by Srinivasan et al. [25] in neonates with meningitis have investigated the utility of cytokines as diagnostic markers in this population. They found that TNF-α, IL-1, IL-6, IL-8, IL-10, and IL-12 levels were significantly elevated in infants with bacterial meningitis. However, none of these cytokines were considered sufficiently accurate for a differential diagnosis, which led to the examination of IL-23, IL-18, and soluble receptor for advanced glycation end products (sRAGE) in the same population [32]. The authors concluded that IL-23 might be a valuable biomarker for the diagnosis of neonatal bacterial meningitis and, when combined with IL-18 and sRAGE, diagnosed meningitis with 100% sensitivity and specificity [32]. Interestingly, relevant to the current report, of the 11 neonates with confirmed meningitis in these studies, there were 4 patients with ventriculoperitoneal shunts in place [25, 32].

To date, no study has examined CSF chemokine and cytokine expression exclusively in patients with CSF shunt infections. Animal models of shunt infection suggest that the inflammatory response in these biofilm infections may be attenuated in comparison to planktonic infections; thus, by extension, the same cytokine and chemokine responses may not be observed in shunt infections as in meningitis [9, 10, 33–35]. One study of shunt infection identified a possible role for soluble membrane attack complex in diagnosis of shunt infection, but this molecule was not able to identify many cases of *S*. *epidermidis* infection, which is the most common cause of shunt infection [36]. Therefore, to characterize potential biomarkers of CSF shunt infection, we evaluated systemic indicators of infection as well as CSF cytokines in a cohort of patients diagnosed with CSF shunt infection.

## Methods

In this retrospective study, we included all patients less than or equal to 21 years of age with culture-confirmed bacterial CSF shunt infection diagnosed between July 1, 2013, and December 31, 2015, at Children’s Hospital and Medical Center (CHMC) in Omaha, Nebraska. Patients were identified by International Classification of Disease, 9th revision coding, and confirmed by positive CSF culture results in accordance with the modified criteria for nosocomial infection of the US Centers for Disease Control and Prevention [15]. CSF culture was performed in the clinical microbiology laboratory at CHMC in accordance with the Clinical Microbiology Procedure Handbook [17]. CSF cell count, differential, glucose, and protein levels were assessed in the clinical laboratory at CHMC in accordance with Clinical & Laboratory Standards Institute protocols [37, 38].

Patients were excluded if their diagnostic CSF culture was performed at another institution or grew yeast or if no CSF culture data was available. All participants were screened for additional inflammatory infection by medical chart review and were excluded if there was an additional inflammatory condition documented in the medical record that would alter inflammatory markers, such as a concomitant viral upper respiratory infection.

Data extracted from the patients’ chart included the following: patient age, peripheral white blood cell (WBC) count, peripheral C reactive-protein (CRP), CSF WBC count and differential, CSF glucose, CSF protein, and CSF culture results. Patient’s peripheral WBC, CRP, CSF WBC and differential, CSF glucose, and CSF protein were included if they were obtained within a 48-h window from the time of the CSF culture.

For those patients with CSF in frozen storage from the same sample that was used for diagnostic culture, additional cytokine analysis was conducted. The CSF was analyzed by multiplex analysis for cytokines and chemokines (Millipore Milliplex Billerica, MA). The multiplex panels included the following: chemokine ligand 2 (CCL2), chemokine ligand 3 (CCL3), fractalkine (CX_3_CL_1_), vascular endothelial growth factor (VEGF), TNF-α, IFN-γ, IL-1β, IL-6, IL-8, IL-10, and IL-17A.

Descriptive statistics were performed using SigmaPlot. A *t* test was used to compare mean cytokine levels in those patients with gram-positive shunt infection compared to those with gram-negative infection. This study was approved by the University of Nebraska Medical Center Institutional Review Board.

## Results

A total of 11 individual patients with 12 separate shunt infections were included in this study (Table 1). Patient ages ranged from 5 weeks to 20 years with a median age of 4 months. All patients had a culture-confirmed ventriculoperitoneal shunt infection. Organisms identified from culture included the following: methicillin-resistant *Staphylococcus aureus* (1), viridans group streptococci (1), coagulase-negative staphylococci (2), *Enterococcus faecalis* (1), *Gemella haemolysans* (1), *Serratia marcescens* (1), *Pseudomonas aeruginosa* (2), *Escherichia coli* (1), *Enterobacter cloacae* (1), and *Stenotrophomonas maltophilia* (1).

**Table 1:** Patient age, CSF parameters, and CSF culture results

Patient number	Age (months)	CRP (mg/dL)	Peripheral WBC (cells/mL)	CSF WBC (cells/mL)	CSF WBC differential (percentage)				CSF protein (mg/dL)	CSF glucose (mg/dL)	CSF culture result	Cytokine analysis performed
					N	L	M	O				
1	36	35.9	17.33	NA	NA				NA	NA	Methicillin-resistant *Staphylococcus aureus*	No
2	5	NA	18.26	NA	NA				NA	NA	Viridans group streptococci	No
3	8	5.1	9.63	1575	98	0	1	0	166	20	*Gemella haemolysans*	No
4	3	17.4	8.54	985	99	1	0	0	600	21	Coagulase-negative staphylococci	Yes
5	24	NA	NA	47	69	18	13	0	114	67	Coagulase-negative staphylococci	Yes
6	240	8.9	29.71	377	86	6	8	0	600	75	*Enterococcus faecalis*	Yes
7	1.25	NA	12.2	NA					NA	NA	*Enterobacter cloacae*	No
7	3	NA	27.57	212	8	52	40	0	NA	NA	*Serratia marcescens*	No
8	3	0.5	13.33	332	26	31	43	0	212	23	*Pseudomonas aeruginosa*	Yes*
9	3	19.1	15.57	1914	80	1	10	9	600	20	*Pseudomonas aeruginosa*	Yes
10	84	27	12.73	24	8	21	68	3	24	61	*Stenotrophomonas maltophilia*	Yes*
11	5	19.9	5.97	28	79	13	6	2	600	48	*Escherichia coli*	Yes

*CRP* C-reactive protein, *WBC* white blood cell count, *CSF* cerebrospinal fluid, *N* neutrophils, *L* lymphocytes, *M* monocytes, *O* other cell type, *NA* data not available

*Not included in analysis for IFN-γ of IL-17A

Of the eight patients with CRP, the median was 18.25 mg/dL (0.5–35.9 mg/dL). All but one patient had a peripheral WBC count performed within 48 h of their CSF culture, and the median WBC was 15.53 × 10^3^ cells/mL (5.97–29.71 × 10^3^ cells/mL). Nine patients had CSF WBC within 48 h of their CSF culture with a median CSF WBC of 332 cells/mL (24–1914 cells/mL). The gram-positive infections appeared to have a neutrophil predominance in the CSF while gram-negative infections had a more varied cellular differential. Eight patients had results for CSF protein and CSF glucose. CSF protein had a median value of 406 mg/dL (24–600 mg/dL), and CSF glucose had a median value of 35.5 mg/dL (20–75 mg/dL).

**Fig. 1:**
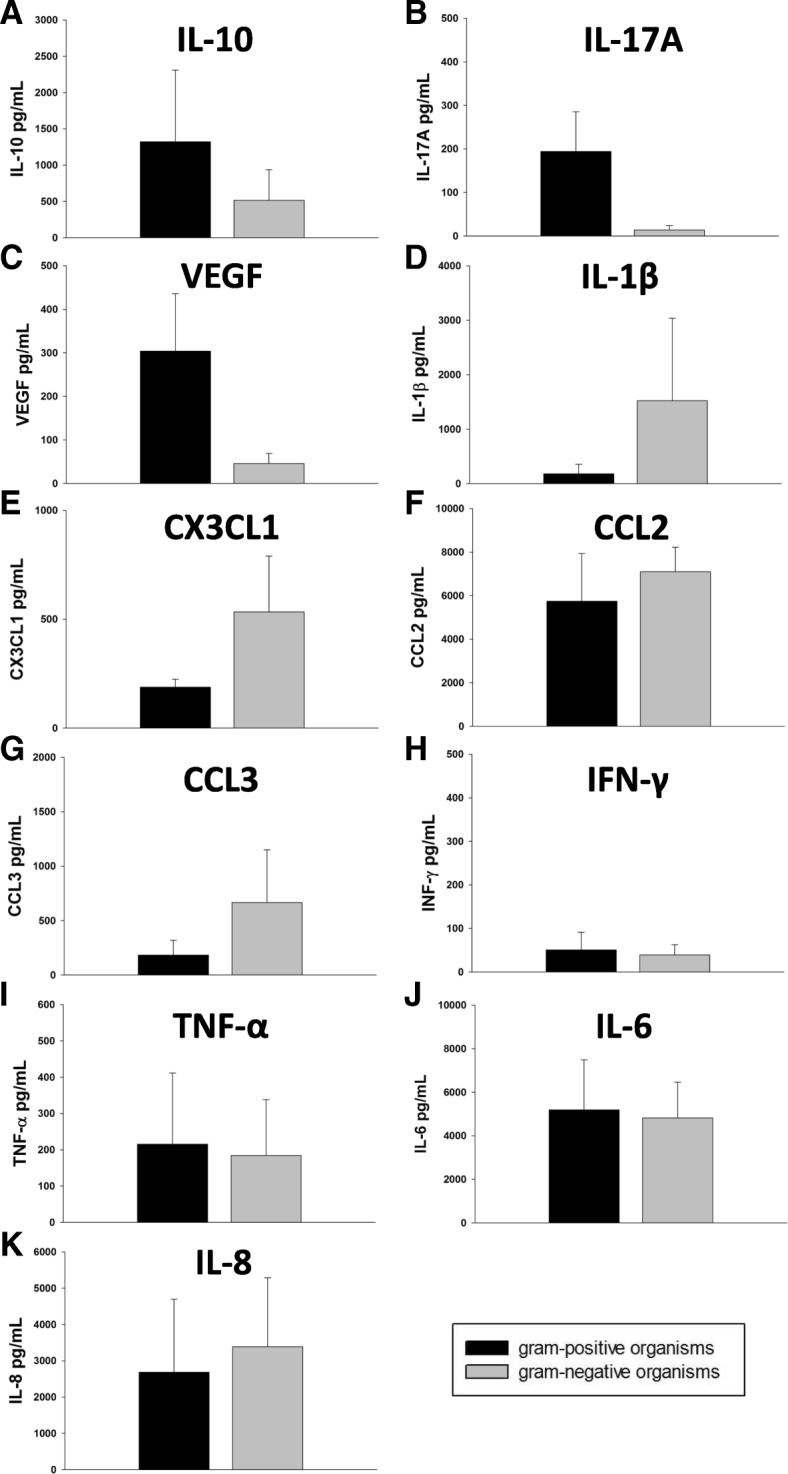
CSF from patients with gram-positive infection has elevated levels of IL-10 (**a**), IL-17A (**b**), and VEGF (**c**) compared to gram-negative infections. Gram-negative shunt infections demonstrate increases in IL-1β (**d**), Cx_3_CL_1_ (E), CCL2 (**f**), and CCL3 (**g**). Gram-positive and gram-negative infections resulted in nearly equivalent levels of INF-γ (**h**), TNF-α (**i**), IL-6 (**j**), and IL-8 (**k**). *n* = 3 patients in gram-positive group, *n* = 2–4 gram-negative group (2 samples had sufficient quantity for measurement of IFN-γ and IL-17A)

Seven of the patients had CSF available for cytokine analysis; of these, three had gram-positive infections and four had gram-negative. While not statistically significant, an interesting trend was observed with CSF from patients with gram-positive infections having higher levels of the anti-inflammatory cytokine IL-10 (1321.17 pg/mL vs 514.49 pg/mL) as well as IL-17A (193.95 pg/mL vs 14.05 pg/mL) and VEGF (303.84 pg/mL vs 45.37 pg/mL) (Fig. 1). In contrast, the gram-negative group had increases in IL-1β (1522.76 pg/mL vs 179.79 pg/mL) and numerous chemokines, including CX_3_CL_1_ (532.93 pg/mL vs 187.95 pg/mL), CCL2 (7094.50 pg/mL vs 5741.00 pg/mL), and CCL3 (665.74 pg/mL vs 183.06 pg/mL) (Fig. 1). Levels of IFN-γ (39.06 pg/mL vs 50.75 pg/mL), TNF-α (184.14 pg/mL vs 215.41 pg/mL), IL-6 (5191.26 pg/mL vs 4816.35 pg/mL), and IL-8 (3390.38 pg/mL vs 2687.22 pg/mL) were similar in both gram-negative and gram-positive infections (Fig. 1).

## Discussion

Prompt diagnosis of shunt infection can allow for earlier treatment, including surgical intervention, potentially averting damaging consequences such as seizures and reduced IQ [39, 40]. However, CSF cultures may be negative due to prior antibiotic treatment or the difficulty of traditional culture in detecting biofilm infections. In these settings, clinicians are forced to use CSF indices and peripheral markers of inflammation to diagnose shunt infection. As observed in our cohort, peripheral leukocytosis is not always present in shunt infection and is very non-specific. CRP elevation was noted in our cohort and in others, but this has been inconsistent in other case studies [20, 21, 41]. CSF leukocytosis is a more specific measure for CSF infection and was observed in this group of patients. However, this can also occur in the setting of uninfected post-surgical inflammation, which presents a significant confounder as the post-surgical period is also the highest risk for shunt infection [42]. CSF protein was elevated in our cohort; unfortunately, prior studies have shown that CSF protein levels cannot distinguish aseptic post-surgical inflammation from shunt infection [42]. While hypoglycorrhachia occurred in our patients, this is again unable to differentiate post-surgical chemical meningitis from shunt infection [42]. In this population where infection most commonly occurs within 30 days of surgery, it is crucial to be able to differentiate post-surgical inflammation from shunt infection. Identification of more specific markers of infection is essential to improve our ability to identify these infections.

Pro-inflammatory mediators have been explored as a diagnostic strategy for bacterial meningitis. Multiple studies have demonstrated elevated levels of TNF-α, IL-6, and IL-8 in the CSF of patients with bacterial meningitis, especially in the pediatric population [22–25, 29, 30]. While there is promising data for TNF-α, IL-6, and IL-8 as diagnostic biomarkers in meningitis, CSF shunt infections are a biofilm rather than planktonic infection which skews the host immune response [10, 12]. Therefore, we examined cytokine/chemokine expression in the CSF of patients with gram-positive and gram-negative shunt infection.

The results of our study demonstrated similar levels of INF-γ, TNF-α, IL-6, and IL-8 in the CSF of patients with both gram-positive and gram-negative CSF shunt infections. This is consistent with results from Srinivasan et al. [25], which demonstrated elevated levels of these mediators in neonates with meningitis, 36% percent of which had a shunt infection. Our data as well as multiple studies in pediatric meningitis demonstrate that TNF-α, IL-6, and IL-8 are present in the CSF of patients with neurologic infection; however, these are likely not good markers for differentiating shunt infection from meningitis [24, 25, 30].

In our cohort, there were trends of a distinct inflammatory response in gram-positive versus gram-negative shunt infections. CSF from patients with gram-positive infections had higher levels of the anti-inflammatory cytokine IL-10 as well as IL-17A and VEGF. Those with gram-negative infection had higher levels of the pro-inflammatory cytokines IL-1β, CX_3_CL_1_ CCL2, and CCL3.

The increase in IL-10 in the gram-positive patients is consistent with a murine model of *S*. *epidermidis* CNS catheter infection [34]. This study demonstrated elevated levels of IL-10 in mice with *S*. *epidermidis* infected compared to sterile catheters [34]. IL-10 is an anti-inflammatory cytokine essential for controlling the production of pro-inflammatory cytokines [43, 44]. It is known to play a role in acute and chronic inflammation in the CNS, and in the setting of CNS catheter infection, its presence may curb the inflammatory environment [34, 45]. Additionally, the presence of IL-10 has been highly associated with other types of staphylococcal infection. Rose et al. [46] demonstrated patients with elevated levels of IL-10 at the time of presentation with *Staphylococcus aureus* blood stream infection had higher mortality. Interestingly, two of the patients included in our cytokine analysis cohort had coagulase-negative staphylococcal infections. The increase in IL-10 may be dampening pro-inflammatory mediators, such as the elevated IL-17A and VEGF seen in these patients, and serving a neuroprotective role. This is being further evaluated in animal models of shunt infection.

In addition to increased IL-10, patients with gram-positive infections had higher levels of IL-17A and a neutrophil predominance in the CSF when compared to patients with gram-negative shunt infection. This is consistent with IL-17’s role as an inducer of neutrophil chemokines, including IL-8, and IL-17’s importance in a murine model of *Staphylococcus aureus* brain abscess [47, 48]. While shunt infections are biofilm rather than planktonic infections like brain abscess, the increased levels of IL-17A and neutrophil predominance in the CSF in our cohort suggest a Th17 response to gram-positive shunt infections in humans may predominate.

In addition to elevated IL-10 and IL-17A, patients with gram-positive shunt infection had elevated levels of VEGF, a potent mediator of vascular permeability associated with cerebral edema and neutrophil migration in the CNS [49–51]. VEGF has been shown to be elevated in a rabbit model of *Streptococcus pneumoniae* meningitis as well as in tuberculous meningitis and other types of bacterial meningitis [52–54]. VEGF may be playing a role in the disruption of the blood-brain barrier in CNS infections, although it is unclear why VEGF is higher in patients with gram-positive shunt infection. This will be explored further in future studies.

Our ability to evaluate whether the gram-negative shunt infections have a Th1, Th2, or Th17 bias was limited due to sample availability. However, the data would suggest a potential skewing towards a Th1 immune response in gram-negative shunt infection with increased monocytes in the CSF of these patients compared to gram-positive infection as well as increase in IL-1β and a modest increase in INF-γ in gram-negative infection. While there is limited data on the type of immune response gram-negative bacteria create in the CNS, studies have demonstrated that gram-negative elicit Th1 immune responses in models of intestinal inflammatory disorders [55, 56].

The CSF patterns reported in this study are likely reflective of differing immune pathways triggered by gram-positive versus gram-negative organisms [23]. Importantly, while chemokine and cytokine profiles have been evaluated as CSF biomarkers of meningitis, evaluation of these markers in the setting of shunt infection has been limited [25, 29, 31, 36]. Our results suggest that the distinct cytokine profiles elicited by gram-positive and gram-negative pathogens show promise as diagnostic tools for CNS shunt infections.

## Conclusions

This pilot study is the first to characterize the CSF cytokine profile in patients with CSF shunt infection and has revealed distinct chemokine and cytokine profiles between gram-negative and gram-positive infections. While there is an interesting trend in the CSF cytokines that could greatly improve diagnosis of CSF shunt infection, there are several limitations with this pilot study. Epidemiologically, coagulase-negative staphylococci is the most common cause of CSF shunt infection, causing approximately two thirds of shunt infections, with *Staphylococcus aureus* as the second most common cause [4, 5, 33]. In contrast, this cohort of patients included a broad range of causative bacteria which did not follow typical epidemiologic patterns. However, our data demonstrate the elevation of chemokines and cytokines in the CSF of patients with shunt infection demonstrating the potential of CSF chemokine and cytokine measurement serving as a diagnostic strategy for shunt infection.

Secondly, in this small cohort, which was not large enough to determine statistical significance, clear trends of chemokines and cytokines were evident in gram-positive versus gram-negative shunt infection. These trends demonstrate a potential Th17-predominant immune response to gram-positive shunt infection and Th1-predominant response in gram-negative shunt infection. Additionally, due to the restrictions of available CSF, there is not a control population as CSF is not obtained from the shunts of well children as there is the possibility of iatrogenic infection. To address these limitations, we are currently developing a rat model of CSF shunt infection to further delineate CSF and serum inflammatory profiles in a controlled manner that will allow for analysis of postoperative versus infective changes. However, these pilot findings serve as a valuable introduction to the potential of CSF chemokines and cytokines as biomarkers for the diagnosis of shunt infection. By improving our ability to diagnose these infections, we can provide earlier effective therapy in a disease process which remains difficult to diagnose and treat.

## Abbreviations

CCL2: Chemokine ligand 2
CCL3: Chemokine ligand 3
CNS: Central nervous system
CRP: C-reactive protein
CSF: Cerebrospinal fluid
CX_3_CL_1_: Fractalkine
IL: Interleukin
INF-γ: Interferon gamma
MCP-1: Monocyte chemoattractant protein-1
MMP-9: Matrix metallopeptidase-9
PCR: Polymerase chain reaction
sRAGE: Soluble receptor for advanced glycation end products
TNF-α: Tumor necrosis factor alpha
VEGF: Vascular endothelial growth factor
WBC: White blood cell
